# Strain assessment in patients with aortic regurgitation undergoing transcatheter aortic valve implantation: case series

**DOI:** 10.1093/ehjcr/ytae261

**Published:** 2024-07-05

**Authors:** Joanna Bartkowiak, Vratika Agarwal, Mark Lebehn, Tamim M Nazif, Isaac George, Susheel K Kodali, Torsten P Vahl, Rebecca T Hahn

**Affiliations:** Department of Medicine, The NewYork-Presbyterian/Columbia University Irving Medical Center, 177 Fort Washington Avenue, New York 10032, USA; Graduate School for Health Sciences, University of Bern, Bern, Switzerland; Department of Medicine, The NewYork-Presbyterian/Columbia University Irving Medical Center, 177 Fort Washington Avenue, New York 10032, USA; Department of Medicine, The NewYork-Presbyterian/Columbia University Irving Medical Center, 177 Fort Washington Avenue, New York 10032, USA; Department of Medicine, The NewYork-Presbyterian/Columbia University Irving Medical Center, 177 Fort Washington Avenue, New York 10032, USA; Department of Medicine, The NewYork-Presbyterian/Columbia University Irving Medical Center, 177 Fort Washington Avenue, New York 10032, USA; Department of Medicine, The NewYork-Presbyterian/Columbia University Irving Medical Center, 177 Fort Washington Avenue, New York 10032, USA; Department of Medicine, The NewYork-Presbyterian/Columbia University Irving Medical Center, 177 Fort Washington Avenue, New York 10032, USA; Department of Medicine, The NewYork-Presbyterian/Columbia University Irving Medical Center, 177 Fort Washington Avenue, New York 10032, USA

**Keywords:** Case report, Aortic regurgitation, TAVI, LV strain, LV remodelling

## Abstract

**Background:**

Limited data exist on strain changes after transcatheter aortic valve implantation (TAVI) in patients with aortic regurgitation (AR).

**Case summary:**

Three patients with AR undergoing TAVI showed an initial reduction in global longitudinal strain (GLS), followed by sustained GLS improvement within the first year.

**Discussion:**

Findings align with those of surgically treated patients with AR. There is a possible superiority of GLS to left ventricular end-diastolic diameter ratio in assessing patients with severe volume overload.

**Learning points**

- Global longitudinal strain (GLS) rises proportionally with stroke volume, anticipating elevated values in severe aortic regurgitation (AR).
- Following transcatheter aortic valve implantation in patients with AR, an initial GLS decrease, followed by ongoing improvement in GLS during the follow-up period, is expected.
- Integrating left ventricular dimensions into GLS calculations holds promise for streamlining assessment in individuals with substantial volume overload.

## Introduction

Global longitudinal strain (GLS) has emerged as an exceedingly sensitive marker of subclinical left ventricular (LV) dysfunction and prognostic indicator in patients with aortic regurgitation (AR).^1^ A worsening of GLS, both before and after surgical aortic valve replacement (SAVR), has been linked to adverse outcomes, even in patients with preserved ejection fraction (EF).^2^

Although SAVR has been the conventional therapeutic approach for severe AR of the native valve, recent advancements in transcatheter devices have enabled the treatment of high-risk or inoperable patients who would otherwise have limited treatment options. The Trilogy System (JenaValve Technology, Irvine, CA, USA) is a pericardial transcatheter aortic valve replacement system designed to treat aortic stenosis (AS) and regurgitation in elderly patients.^3,4^ The distinctive attribute of the valve includes a triad of locators facilitating the attachment to the native aortic valve cusps, ensuring commissural alignment (see Supplementary material online, *Video S1*). This anchoring mechanism relies less on radial force, thereby promoting stable valve positioning, even in the presence of minimal or no aortic calcification. Furthermore, the prosthesis securely grasps the native cusps, mitigating the risk of coronary obstruction.

**Figure 1:**
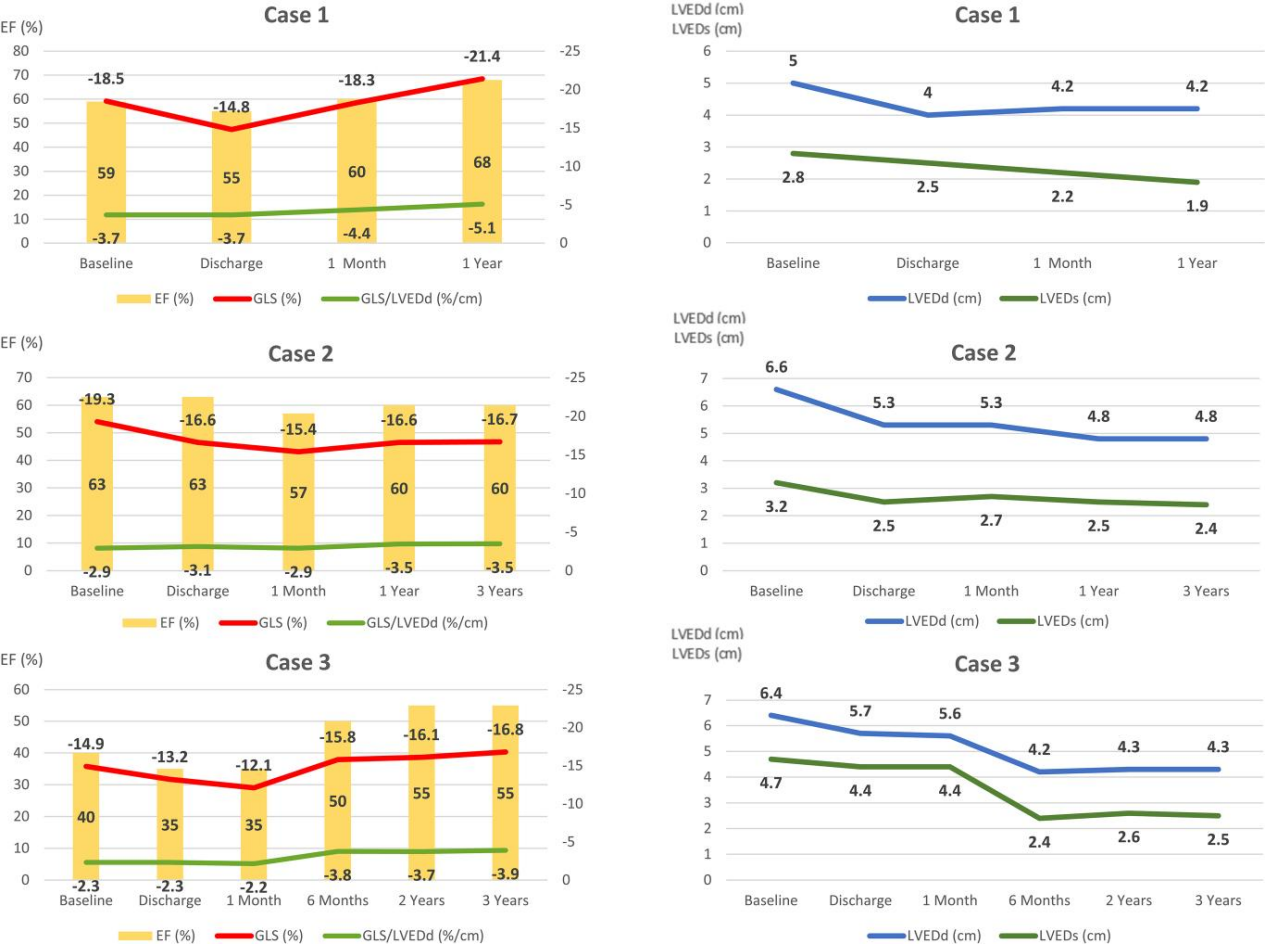
Changes in left ventricular function (left panels) and geometry (right panels) over time are shown for all three patients. Following the procedure, all patients initially experienced a slight worsening of global longitudinal strain (upper line in the left panels) and ejection fraction (bars), as well as a reduction of left ventricular end-diastolic (upper line in the right panels) and end-systolic (lower line in the right panels) dimensions. The global longitudinal strain to left ventricular end-diastolic diameter ratio is shown in the left panels (lower line in the left panels).

The prospective ALIGN-AR trial investigating the Trilogy valve met the predefined 30-day performance goal for safety outcomes (26.7%, *P* for non-inferiority <0.0001), as well as the 12-month performance goal for the efficacy outcome of all-cause mortality (7.8%, *P* for non-inferiority <0.0001).^5^ However, the impact of transcatheter aortic valve implantation (TAVI) on LV function and its prognostic implications in high-risk AR patients remains unclear. In this study, we present three cases of patients with AR who underwent TAVI using the JenaValve Trilogy System and report the corresponding changes of GLS, LVEF, and LV dimensions. GLS tracings were performed using dedicated post-processing software (TomTec Arena, TomTec, Unterschleißheim, Germany) and manually corrected in all cases. All patients presented in this case series were deemed inoperable by a multidisciplinary heart team. The baseline and procedural characteristics of all patients are listed in *Table 1*, and the changes in transthoracic echocardiographic (TTE) LV function parameters over time are displayed in *Figure 1*. A post-TAVI evaluation showed normal transcatheter valve function for all patients (*Table 1*).

**Table 1:** Baseline and procedural characteristics

	Case 1	Case 2	Case 3
Baseline characteristics			
Age (years)	83	72	86
Symptoms	NYHA II	NYHA II	NYHA III
STS score (%)	2.8	3.8	4.5
EuroScore II	3.8	8.9	5.0
LVEDd (cm)	5.0	6.6	6.4
LVESd (cm)	2.8	3.2	4.7
IVS (cm)	0.7	1.1	1.0
PW (cm)	0.8	1.0	1.0
EF (%)	59	63	40
Aortic stenosis	Mild	None	None
Max gradient (mmHg)	43	26	10
Mean gradient (mmHg)	20	12	5
AVA (cm^2^)	1.9	2.5	3.6
Aortic regurgitation	Severe	Severe	Severe
Aetiology	Degenerative	RCC prolapse	NCC prolapse
PHT (ms)	390	x	314
Vena contracta (mm)	7.1	8.0	6.9
RegVol (mL)	59	75	90
RegFrac (%)	46	52	69
EROA (mm^2^)	22	27	40
3D VCA (mm^2^)	41	36	38
3D reg vol (mL)	69	99	89
Flow reversal in descending aorta	Yes	Yes	Yes
Procedural characteristics			
Implanted valve	23 mm JV	27 mm JV	25 mm JV
Post-procedural peak aortic gradient (mmHg)	3.5	2.5	2.5
Post-procedural peak aortic gradient (mmHg)	7	5	5
Post-procedural aortic valve area (cm^2^)	2.2	3.3	3.0
Residual regurgitation	Trace paravalvular AR	Trace paravalvular AR	No AR
NYHA last follow-up	NYHA II	NYHA I	NYHA I

3D, three-dimensional; AVA, aortic valve area; EF, ejection fraction; EROA, effective regurgitant orifice area; IVS, interventricular septum; LVEDd, left ventricular end-diastolic diameter; LVESd, left ventricular end-systolic diameter; NCC, non-coronary cusp, NYHA, New York Heart Association Classification; PHT, pressure half-time; PW, posterior wall; RCC, right coronary cusp; RegFrac, regurgitation fraction; RegVol, regurgitation volume; VCA, vena contracta area.

## Summary figure

**Figure:**
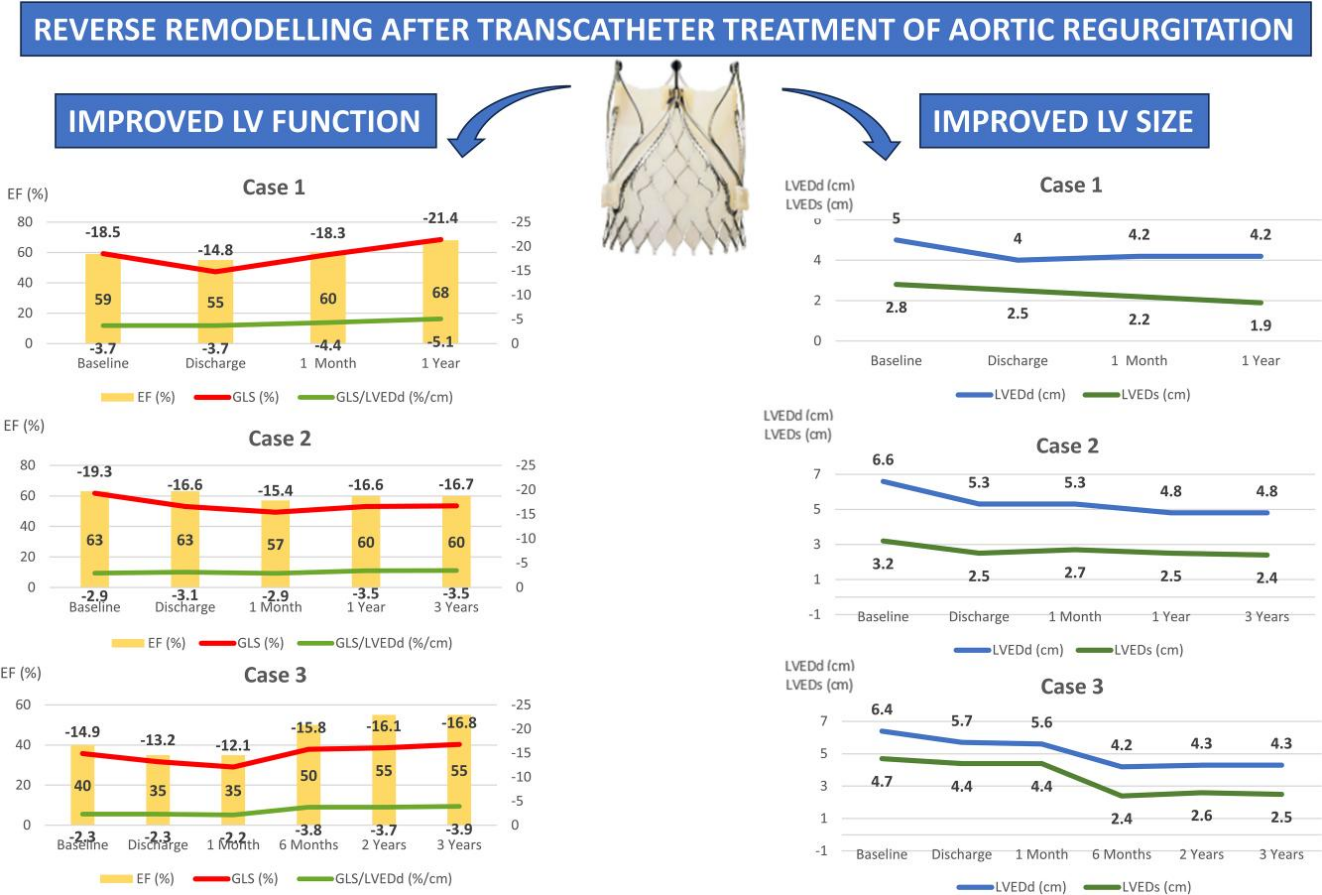


## Case 1

**Figure 2:**
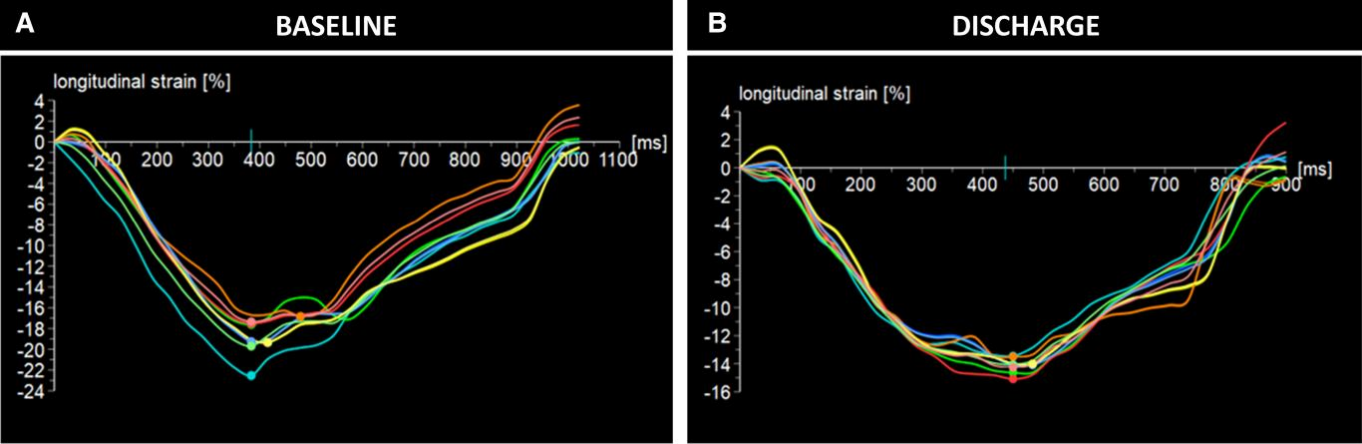
Baseline (*A*) and discharge (*B*) strain values of Patient 1 showing a slight worsening of global longitudinal strain, likely attributed to the acute increase in afterload resulting from transcatheter aortic valve implantation.

An 83-year-old female with a medical history of ischemic stroke and Takotsubo cardiomyopathy, initially diagnosed with an LVEF ranging between 25 and 30%, was referred for the evaluation of severe AR, which was incidentally discovered during the diagnostic workup of stroke. Baseline TTE (*Figure 2A*, Supplementary material online, *Video S2A*) confirmed severe degenerative AR with mild AS and preserved LV function [LV end-systolic diameter (LVESd) = 2.8 cm, EF 59%, and GLS −18.5%]. The patient underwent successful treatment with a 23 mm JenaValve without any complications. Following the procedure, the discharge TTE showed no relevant change in LVEF; however, there was a clear reduction in both LV end-diastolic diameter (LVEDd) and LVESd, and GLS to −14.8% (*Figure 2B*, Supplementary material online, *Video S2B*). At the 1-month follow-up, the GLS returned to the baseline values and normalized at the 1-year follow-up (*Figure 1*).

Comment: In this case, treatment of AR results in an immediate reduction in absolute GLS at discharge, accompanied by a reduction in LV dimensions. Although this would typically be seen as a reduction in mechanical deformation, the GLS to LV end-diastolic diameter (LVEDd) ratio, in fact, did not significantly change at discharge and continued to improve during follow-up. The subsequent normalization of the GLS during follow-up suggests the absence of irreversible LV fibrosis.

## Case 2

**Figure 3:**
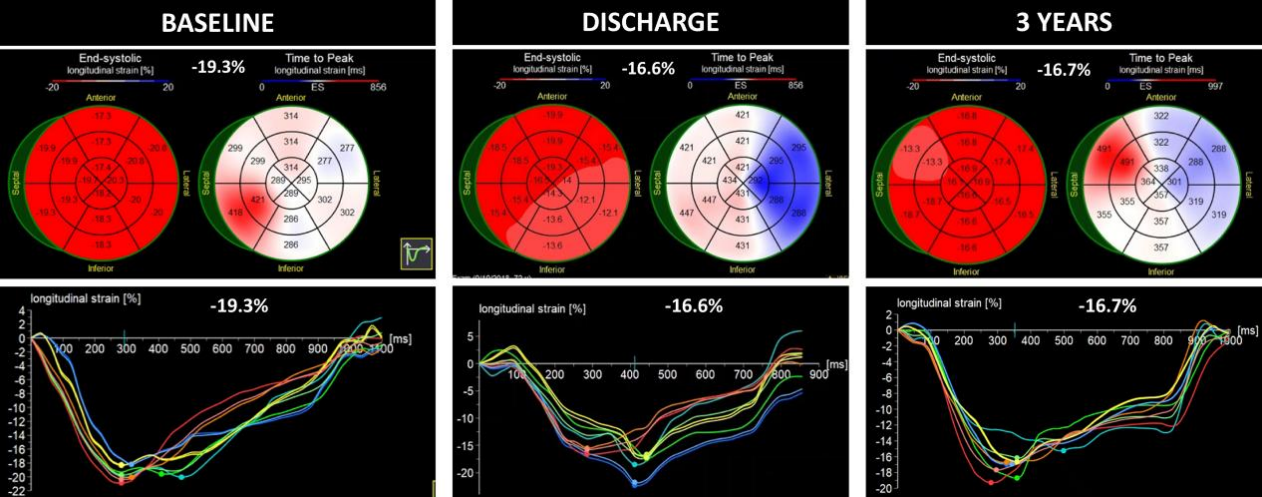
Changes in the global longitudinal strain of Patient 2 showing an initial decrease in global longitudinal strain (*Figure 2A*), which persisted until the last follow-up (*Figure 2C*), despite normal ejection fraction and a well-functioning aortic valve prosthesis.

A 72-year-old male patient with a history of hypertension, type II diabetes mellitus, coronary artery disease, and stage IV chronic kidney disease presented to our clinic seeking management for symptomatic severe AR [New York Heart Association (NYHA) Class III]. The baseline TTE revealed a prolapse of the right coronary cusp, without stenosis (see Supplementary material online, *Video S3*). The LV was dilated with an LVEDd of 6.6 cm, an LVESd of 3.2 cm, and preserved systolic function (an EF of 63% and a GLS of −19.3%, *Figure 3A*). The patient underwent a successful TAVI with a 27 mm JenaValve without any complications. The discharge TTE showed no significant change in the EF, but there was a reduction in LV dimensions and GLS (*Figure 3B*). Throughout the follow-up period, there was continued LV remodelling with a reduction in the LVEDd and LVESd (*Figure 1*). Although the GLS improved following the first post-TAVI follow-up, it did not return to baseline value (*Figure 3C*), which is suggestive of possible irreversible myocardial fibrosis.

Comment: The normal baseline GLS may suggest the absence of significant myocardial structural changes; however, in the setting of a dilated LV, a high GLS may be a reflection of a high preload. The GLS:LVEDd ratio is much lower than that of Case 1, which may suggest abnormal deformation for a dilated ventricle. Despite the reduction in the GLS during the follow-up, the GLS:LVEDd ratio shows no significant change either at discharge or at 6 months, with an improvement in mechanical deformation at 1 and 3 years of follow-up. Previous surgical studies have demonstrated that the failure to achieve GLS normalization up to 1 year following SAVR is linked to unfavourable long-term outcomes even if the EF is preserved.^2^ On the other hand, a continuous improvement of the GLS:LVEDd ratio resulting from a reduction of the LV dimensions indicates LV reverse remodelling associated with a risk reduction of major adverse cardiovascular events following SAVR for AR.^6^

## Case 3

An 86-year-old female with a medical history of breast cancer (treated surgically and with radiation in 1987), pulmonary hypertension, chronic heart failure with reduced EF, and severe AR presented with worsening fatigue and dyspnoea (NYHA Class III). The baseline TTE revealed marked LV dilatation with eccentric hypertrophy, LVEF of 40% with global hypokinesis, and a significantly reduced GLS (see Supplementary material online, *Video S4A*). There was a primary AR resulting from a prolapse of the non-coronary cusp. The patient underwent a successful implantation of a 25 mm JenaValve without any complications. The discharge TTE demonstrated a small reduction in the EF (Δ5%) and strain values (Δ1.7%; see Supplementary material online, *Video S4B*). Subsequent TTE assessment indicated a further worsening of the GLS at 1 month, followed by a gradual and continuous improvement of all parameters starting from the 6-month follow-up. Although the GLS had improved compared with baseline, it did not reach the normal values (*Figure 1*, Supplementary material online, *Video S4C*).

Comment: This patient has the lowest absolute value of GLS, accompanied by the lowest baseline LVEF and lowest GLS:LVEDd. In this setting, post-TAVI improvement was slower than that in the previously mentioned patients, although ongoing LV reverse remodelling and improvement in the LVEF, GLS and GLS:LVEDd was seen during the longer-term follow-up. A persistent reduction in the GLS, despite the normalization of the EF during the follow-up, suggests the presence of irreversible myocardial fibrosis, possibly induced by past radiation exposure and AR.

## Discussion

Numerous studies have shown the prognostic importance of strain measurements for assessing myocardial function in aortic valve disease.^1,7,8^ However, GLS is influenced by load conditions, and therefore, different cut-off values have been proposed depending on the underlying valve pathology. For example, in AS, the risk increases below an absolute GLS value of <12.1%^7^ and in mitral regurgitation with a GLS of <18.1%.^8^ Currently, there is no consensus on the normal cut-off values for strain in patients with AR, but studies consistently indicate an elevated risk of adverse events with GLS values reaching below the range of 17–19%.^9^

In patients with AR undergoing SAVR, an initial acute reduction in strain was observed, followed by subsequent strain normalization within 3–12 months post-SAVR.^2^ Both diminished baseline strain values and the failure of the strain to normalize after the procedure were associated with adverse clinical outcomes.^2^ There are no studies describing strain changes occurring after TAVI in patients with AR, primarily due to the absence of dedicated transcatheter devices for pure AR treatment. Recently, the JenaValve system received a Conformité Européenne mark for transcatheter treatment of AR based on the ALIGN-AR trial. This study provided valuable insights into LV remodelling in patients with AR undergoing TAVI, showing significant improvements in the LVESd, LV end-systolic volume, LV mass, and LV mass index (*P* for all <0.0001) throughout a 1-year period, which aligns with our observations.

GLS represents the overall deformation of the LV and is indicated by the change in segment length during systole.^10^ Because strain ignores the pre- and afterload under which the fibres have to shorten, it remains a load-dependent surrogate parameter of LV function.^11^ Because ‘wall stress’ depends on chamber geometry, as described by LaPlace’s law, more dilated, thin-walled ventricles may display greater wall stress and lower deformation, irrespective of actual mechanical myocardial dysfunction. This may be particularly relevant when assessing LV function in patients with AR. A deformation index, normalizing the deformation parameters to LV volumes, has been previously suggested to correct for the volume dependency of deformation.^12,13^ If volume overload is present, strain remains normal or increases masking subclinical LV dysfunction. As suggested in previous studies,^12,13^ a parameter accounting simultaneously for LV strain and geometry would better reflect true LV contractility.

The value of normalizing LV strain for LV size may be supported by Vollema *et al*.,^14^ who studied patients undergoing SAVR for AS or AR. They found that after surgery, both groups of patients showed LV mass regression, although a more pronounced decline was seen in patients with AR.^14^ In this study, an improvement in LV GLS was also observed in both patient groups, but it was characterized by an initial decline in patients with AR, while LV GLS in patients with AS remained initially stable. The changes in LV volumes following SAVR or TAVI could explain the drop in the GLS, which may not reflect a change in structural function but rather reverse remodelling. Our patients showed an immediate post-TAVI fall in the degree of the GLS but a ‘stable’ GLS:LVEDd with a gradual improvement over the follow-up period, which is more consistent with a stable (or improved) EF and favourable LV reverse remodelling. Whether the GLS:LVEDd ratio can predict outcomes or is a better marker of baseline LV structural changes, requires validation.

## Supplementary Material

ytae261_Supplementary_Data

## Data Availability

The data underlying this article cannot be shared publicly due to disclosure of Protected Health Information, as defined in the Health Insurance Portability and Accountability Act of 1996 and its implementing regulations (‘HIPAA’). The data will be shared on reasonable request to the corresponding author.

## Lead author biography

Dr Joanna Bartkowiak is a postdoctoral fellow at the NewYork-Presbyterian/Columbia University Irving Medical Center, USA and at the Graduate School for Health Sciences at the University of Bern, Switzerland. She received her medical degree from the University of Medical Sciences in Poland and completed her residency in general cardiology at the University Hospital of Bern in Switzerland. Her areas of expertise include interventional cardiology, valvular heart disease, and cardiac imaging.

## Supplementary material

**Consent:** The authors confirm that written informed consent for submission and publication of this case report including image(s) and associated text has been obtained from the patient in line with COPE guidance.

**Funding:** None declared.
